# The Medicinal Mushroom *Ganoderma*: A Review of Systematics, Phylogeny, and Metabolomic Insights

**DOI:** 10.3390/jof12010058

**Published:** 2026-01-12

**Authors:** Gideon Adotey, Abraham Quarcoo, Mohammed Ahmed Gedel, Paul Yerenkyi, Phyllis Otu, Abraham K. Anang, Laud K. N. Okine, Winfred S. K. Gbewonyo, John C. Holliday, Vincent C. Lombardi

**Affiliations:** 1 Science Laboratory Department, Accra Technical University, Barnes Road, Accra P.O. Box GP 561, Ghana; 2 Noguchi Memorial Institute for Medical Research (NMIMR), University of Ghana, Accra P.O. Box LG 54, Ghana; 3 Department of Biochemistry, Cell and Molecular Biology (BCMB), University of Ghana, Accra P.O. Box LG 54, Ghana; 4 Aloha Medicinals Inc., Carson City, NV 89706, USA; 5 Department of Microbiology and Immunology, School of Medicine, University of Nevada, 1664 N Virginia St. MS 0320, Reno, NV 89523, USA

**Keywords:** *Ganoderma*, molecular taxonomy, phylogenetics, ITS sequencing, molecular barcoding, metabolomics, triterpenes, polysaccharides, pharmacological potential, Reishi, Lingzhi, Africa, species diversity, quality control

## Abstract

*Ganoderma* is a genus of medically significant fungi, that is used in traditional medicine and is increasingly incorporated into modern nutraceuticals and pharmaceuticals. Accurate species identification and product standardization remain major challenges due to morphological plasticity and cryptic diversity. This review articulates current advances in *Ganoderma* systematics, phylogenetics, and metabolomics, with an emphasis on molecular identification strategies and chemical profiling. Internal transcribed spacer (ITS) sequencing has substantially improved species delineation compared with morphology alone, but its resolving power is limited in closely related species complexes, necessitating complementary multilocus approaches. Advances in metabolomics, and LC-MS- and HPLC-based profiling of triterpenes and polysaccharides, have enhanced species discrimination, chemotaxonomic resolution, and quality control of commercial products. Integrating molecular barcoding with metabolomic fingerprints provides a more robust framework for classification, pharmacological evaluation, and standardization. This review also highlights significant geographic knowledge gaps, particularly in Africa, where molecular and metabolomic data remain scarce despite high species diversity.

## 1. Introduction

*Ganoderma* is a genus of medicinal fungi historically used in traditional Eastern medicine, and is particularly common in China and Japan, where it is highly regarded for its immunomodulatory, hepatoprotective, anticancer, and anti-inflammatory properties [1,2]. Among its bioactive constituents, triterpenes, polysaccharides, glucans, and glycoproteins are consistently associated with these pharmacological effects [2,3]. Increasing global demand for complementary and alternative therapies has driven the incorporation of *Ganoderma*-derived compounds into nutraceuticals, functional foods, and pharmaceuticals [4].

Over the last few decades, research on *Ganoderma* has significantly increased, well beyond its therapeutic potential, to encompass its genetic, taxonomic, and metabolic diversity. Accurate species identification and characterization have become critical to ensuring the consistency, efficacy, and safety of *Ganoderma*-based products. In the past, scientists classified species within the *Ganoderma* genus primarily by focusing on physical traits, like the size of the basidiospores, or the shape and structure of the cap (pileus), as well as the texture of the spore walls. However, these features can vary significantly depending on factors such as the specimen’s age, the type of substrate it grows on, and the surrounding environmental conditions. Because of this variability, relying solely on morphology has proven problematic when trying to delineate species [5,6]. Consequently, ambiguity and misclassification have hindered *Ganoderma* taxonomy.

The emergence of molecular phylogenetics has significantly transformed fungal taxonomy, allowing for more accurate species identification through the use of genetic barcoding. The internal transcribed spacer (ITS) region of ribosomal RNA has become a widely recognized barcode for fungi identification because of its high variability between species and its ease of amplification [7]. In addition to ITS, other genetic markers, such as the nuclear large subunit (nLSU), small subunit (nSSU), translation elongation factor 1-alpha (EF1-α), and RNA polymerase II subunit (RPB2), have further improved phylogenetic resolution and identification due to their high interspecific variability and ease of amplification [8].

**Figure 1:**
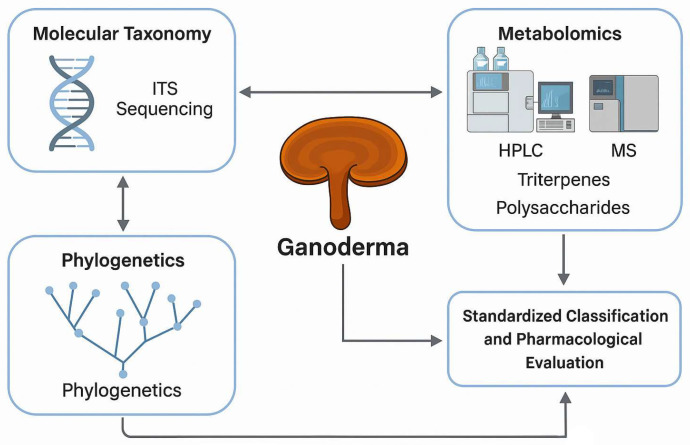
Integrated framework for the molecular taxonomy, phylogenetics, and metabolomic profiling of *Ganoderma* for standardized classification and pharmacological evaluation.

In parallel, metabolomics has become essential for elucidating the biochemical composition of *Ganoderma*. Analytical platforms such as high-performance liquid chromatography (HPLC), mass spectrometry (MS), and nuclear magnetic resonance (NMR) spectroscopy are commonly employed to identify and quantify key secondary metabolites, including ganoderic acids and β-glucans [9]. These compounds play a central role in the pharmacological activity of *Ganoderma*, contributing to its immunostimulatory, antioxidant, and anticancer effects [10,11]. Comprehensive metabolite profiling is, therefore, crucial for product standardization, quality control, and validation of therapeutic efficacy (Figure 1).

While recent progress has expanded our understanding of *Ganoderma*, notable gaps still exist, especially in underexplored areas like sub-Saharan Africa. While several species from this region have been described, studies into the molecular or metabolomic profiles are limited [11]. Moreover, this geographic underrepresentation has hindered global efforts to catalog biodiversity and develop regionally tailored therapeutics.

This review provides an integrative synthesis of the state-of-the-art on *Ganoderma* systematics, phylogenetics, and metabolomics with an emphasis on the application of molecular identification techniques. Specifically, we explore the phylogenetic relationships within the genus and highlight advances in metabolite profiling using modern analytical tools. Methodological limitations, regional research gaps, and future priorities are also discussed, with particular focus on Africa, where emerging studies offer promising new directions.

## 2. Materials and Methods

### 2.1. Literature Search Strategy

This review was made based on a comprehensive survey of the peer-reviewed literature on *Ganoderma* systematics, phylogenetics, and metabolomics. Primary literature searches were conducted using PubMed and Google Scholar, covering publications up to December 2025. Search terms included combinations of *Ganoderma*, *Ganoderma lucidum*, molecular taxonomy, ITS, multilocus phylogeny, metabolomics, triterpenes, polysaccharides, pharmacological activity, and Africa. Reference lists of relevant articles were also screened to identify additional studies. Only peer-reviewed publications written in English were considered.

### 2.2. Use of Artificial Intelligence Tools

During the preparation of this review, ChatGPT (version 5.0; OpenAI) was used to help formulate the initial manuscript outline, generate draft tables and schematic figures, perform grammatical editing, and assist in identifying and verifying references. Artificial intelligence (AI) tools were not used to generate original scientific interpretations or make substantive scientific conclusions. All content generated with AI assistance was critically reviewed, edited, and validated by the authors, who take full responsibility for the accuracy, originality, and integrity of the final manuscript.

## 3. Molecular Taxonomy and Systematics

### Systematics of Ganoderma with Emphasis on Molecular Identification Methods

The genus *Ganoderma*, recognized for both its medicinal significance and ecological functions, has long presented taxonomic challenges. Early classification systems relied primarily on morphological traits, such as basidiospore shape and size, pileus coloration, and spore ornamentation. However, these features are phenotypically plastic and are influenced by environmental conditions, developmental stage, and substrate specificity, resulting in frequent misidentifications [6]. Even basidiospore morphology, once considered diagnostic, has shown limited interspecific variation and reduced discriminatory power [12]. As a result, the taxonomy of *Ganoderma* has been in a state of considerable ambiguity.

The advent of molecular systematics has substantially improved species delineation by providing objective, high-resolution tools that are less susceptible to environmental variability. Among these, the ITS region of the nuclear ribosomal RNA operon has become the standard barcode for fungal identification, including within *Ganoderma* [13,14]. ITS sequencing has facilitated the resolution of cryptic species, clarified evolutionary relationships, and enhanced the accuracy of species-level classification.

Multiple studies have supported the use of the ITS region in conjunction with other loci, such as the nLSU and nSSU rRNA genes, to improve phylogenetic resolution [15,16]. The high interspecific variability of the ITS region has made it especially valuable for distinguishing morphologically similar taxa [17]. However, ITS-based identification has limitations; in some species complexes, ITS sequences alone do not provide sufficient resolution, necessitating supplementary genetic markers [18]. Although ITS remains the primary fungal barcode, its resolving power varies across *Ganoderma* clades; a comparison of commonly used molecular markers highlights differences in taxonomic resolution, amplification success, and clade-specific utility (see Table 1).

The ITS2 subregion has gained attention as a potentially advantageous barcode due to its shorter sequence length and relatively rapid evolutionary rate, which together facilitate efficient amplification and discrimination among closely related taxa [23]. Because ITS2 evolves faster than the flanking 5.8S and LSU regions, it can capture recent divergence events while remaining short enough to reduce alignment ambiguity and sequencing errors. Additionally, its conserved secondary structure allows for improved homology assessment in comparative analyses [21]. However, despite these advantages, ITS2 alone may lack sufficient phylogenetic signal for resolving deeper relationships or complex species groups, and its utility remains highly clade-dependent. Consequently, ITS2 is best applied as a complementary marker rather than a replacement for full ITS or multilocus approaches [27].

The need for molecular tools is especially pressing in biodiverse but undercharacterized regions such as sub-Saharan Africa. In many parts of the continent, *Ganoderma* species have been identified almost exclusively through macroscopic traits, with minimal molecular confirmation [20]. For example, taxonomic surveys in Ghana have largely relied on morphology alone, raising concerns about the reliability of these identifications [11]. Incorporating molecular barcoding and phylogenetic analysis, particularly polymerase chain reaction (PCR)-based techniques, markedly improve the accuracy of species identification and biodiversity assessments in these regions.

Beyond taxonomy, molecular identification methods have practical applications in agriculture and forestry. Several *Ganoderma* species are pathogenic to economically important crops such as cocoa, cashew, and coffee. Molecular diagnostics provide rapid, cost-effective, and accurate tools for early detection and species-level identification, which are essential for the timely management of plant disease outbreaks [12].

In summary, while traditional morphological approaches have laid the foundation for understanding *Ganoderma* diversity, they are increasingly being supplanted by molecular methods that offer greater precision, reproducibility, and scope. The combined use of ITS, ITS2, and additional loci constitutes a robust framework for resolving taxonomic ambiguities and uncovering cryptic species. As molecular technologies become more widely accessible, they are expected to play a pivotal role in advancing both fundamental mycological research and the applied sciences related to *Ganoderma*, particularly in regions where research remains limited but potential biodiversity is high.

## 4. Phylogenetic Advances in Ganoderma Research

### 4.1. Ganoderma Phylogenetics: Molecular Insights and Advancements

**Figure 2:**
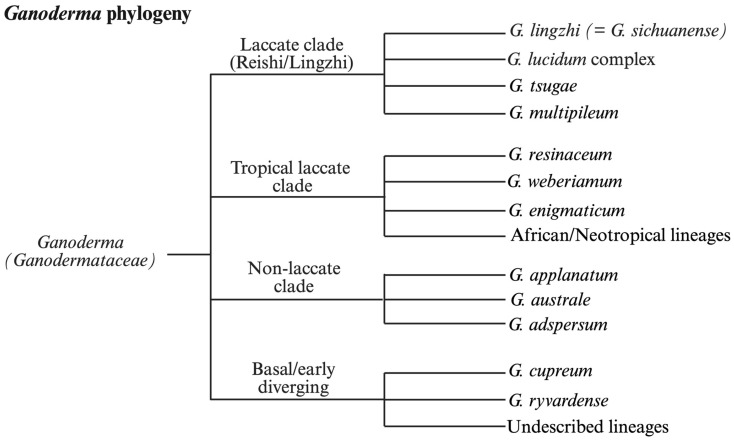
Representative multilocus phylogeny of the genus Ganoderma illustrating major clades recovered across ITS, EF1-α, RPB2, and nLSU datasets. The tree highlights the separation of laccate and non-laccate lineages, resolution of the *G. lucidum* species complex, and the presence of undercharacterized tropical and African clades. Phylogenetic relationships are compiled from prevously published multilocus analyses rather than newly generated sequence data.

Over the past several decades, phylogenetic studies of *Ganoderma* have undergone a significant transformation, driven by advances in molecular biology. Traditional taxonomy, based on macroscopic and microscopic features, has proven inadequate for resolving closely related species within the genus. As a result, researchers have increasingly adopted DNA sequencing and molecular phylogenetic tools to clarify species boundaries and evolutionary relationships (Figure 2).

One of the earliest pivotal studies in *Ganoderma* phylogeny was conducted by Moncalvo et al. [20], who analyzed sequences from the ITS and the 25S rRNA gene. Their work demonstrated that the ITS region could effectively differentiate most species, though it was insufficient to resolve the *G. tsugae* complex. The D2 domain of the 25S rRNA gene proved useful at higher taxonomic levels, and its combination with ITS sequences supported the monophyly of the subgenus *Elfvingia*. This study laid the groundwork for multilocus approaches in *Ganoderma* systematics.

Subsequent research has confirmed that ITS sequencing remains a foundational tool for species delimitation, especially when combined with multiple loci. Zhou and colleagues conducted a multilocus phylogenetic analysis on 32 collections of the *G. lucidum* complex using ITS, tef1α, rpb1, and rpb2. This study identified three distinct genetic clades (A, B, and C) within the complex, none of which corresponded strictly to geographic origin [25]. These findings underscore the extensive taxonomic complexity of *Ganoderma*, revealing that morphological classification alone often fails to accurately distinguish genetically divergent taxa due to overlapping features. A complementary ITS-focused study further demonstrated that ITS1 provided better geographic clustering than ITS2 among *G. lucidum* strains from global origins [22]. However, ITS2 alone was insufficient for fine-scale resolution, highlighting ITS limitations in detecting closely related but distinct lineages.

Xing et al. employed a multilocus phylogenetic framework combining ITS, EF1-α, and RPB2 sequences to resolve species boundaries within the *Ganoderma lucidum* complex [28]. Phylogenetic trees were reconstructed using maximum likelihood (ML) and Bayesian inference (BI) approaches, which yielded congruent topologies. The novel lineage corresponding to *Ganoderma aridicola* formed a well-supported monophyletic clade, with ML bootstrap values exceeding 70% and Bayesian posterior probabilities ≥0.95, supporting its recognition as a distinct species. This study illustrates how multilocus datasets outperform ITS alone in resolving cryptic taxa within *Ganoderma* species complexes. Similar multilocus strategies (typically ITS + EF1-α ± RPB2 or nLSU) have since become standard in *Ganoderma* systematics, providing improved resolution at both shallow and deep phylogenetic levels. He et al. used multilocus phylogenetics in Yunnan Province to describe two novel species, *G. dianzhongense* and *G. esculentum*, based on their genetic divergence from known taxa [29]. These examples demonstrate the power of multilocus strategies for uncovering cryptic diversity and refining species concepts.

### 4.2. Phylogenetic Tools for Product Authentication

Molecular phylogenetics has also become essential for verifying the identity of commercial *Ganoderma* products. Gunnels et al. used ITS-based phylogenetic analysis to confirm the presence of *G. lingzhi* in dietary supplements, supporting the use of this marker for quality assurance in the nutraceutical sector [19]. Liao et al. further employed DNA barcoding to assess strain-level variation in cultivated *G. lucidum* from China and Europe [24]. Their findings revealed significant genetic divergence, suggesting that some commercial *G. lucidum* may consist of multiple cryptic species, a concern with implications for product efficacy and standardization.

Regional molecular studies continue to expand the known diversity of *Ganoderma*, particularly in previously understudied areas. Kinge and coworkers employed ITS and mtSSU sequencing to characterize Cameroonian isolates, identifying eight phylogenetic species, three of which were assigned to named taxa (*G. ryvardense*, *G. cupreum* and *G. weberianum*) while the remaining five lineages did not match any described species [30]. In Egypt, El-Fallal et al. identified *G. resinaceum* and a distinct lineage referred to as *Ganoderma* sp. EGDA from fig and citrus trees using ITS and 5.8S rRNA data [31]. Taken together, these studies emphasize the importance of applying molecular tools globally to revise classifications and uncover undocumented fungal diversity.

A recent revision of *Ganoderma* systematics by Du et al. resolved a long-standing nomenclatural ambiguity by demonstrating that *G. lingzhi* is a later synonym of *G. sichuanense* [26]. Their study, based on phylogenetic comparisons of type specimens, exemplifies the need for molecular verification in taxonomic revision. Sun et al. further employed a six-locus dataset, including ITS, nLSU, EF1-α, and RPB2, to clarify relationships within the Ganodermataceae [16]. Their work showed that multilocus data outperform ITS alone in resolving polytomies and identifying novel species, advocating for broader use of these techniques in future studies.

These phylogenetic clarifications have important implications beyond nomenclature. Accurate species delimitation establishes the necessary framework for species-specific chemical characterization, as metabolite profiles are increasingly recognized to vary among closely related *Ganoderma* taxa. Without robust phylogenetic resolution, metabolomic datasets risk conflating chemically distinct species under a single name, thereby obscuring biologically meaningful variation. Consequently, advances in molecular systematics directly enable more precise metabolomic profiling and interpretation.

In summary, molecular phylogenetics has become central to *Ganoderma* research, informing taxonomy, product authentication, and biodiversity discovery. While ITS remains a foundational marker, multilocus sequencing offers superior resolution, particularly in species complexes and cryptic groups. Continued adoption of these tools is expected to drive a comprehensive reevaluation of *Ganoderma* taxonomy worldwide.

## 5. Metabolomic Profiling and Bioactive Compounds

### 5.1. Ganoderma Metabolomics: Analytical Approaches for Characterization and Quality Control

The growing global demand for *Ganoderma*-based products has spurred interest in profiling its bioactive constituents. Metabolomics, defined as the comprehensive analysis of metabolites within a biological system, has become a critical tool for elucidating the therapeutic potential and ensuring quality control of *Ganoderma* species. Advanced analytical platforms such as liquid chromatography–mass spectrometry (LC-MS), high-performance liquid chromatography (HPLC), and NMR spectroscopy allow for the precise identification and quantification of secondary metabolites, particularly triterpenes and polysaccharides [32]. Given the chemical diversity of *Ganoderma* metabolites, different analytical platforms are required to resolve specific classes of bioactive compounds; a comparative overview of commonly applied metabolomic approaches is provided in Table 2.

### 5.2. LC-MS-Based Triterpene Profiling

Triterpenes, especially ganoderic acids, are among the most pharmacologically active compounds in *Ganoderma*. In 2012 Qi et al. described an LC-MS method for the simultaneous detection of 14 ganoderic acids that utilized previously described analytes as standards [33]. This targeted profiling approach is valuable for quality control in commercial production, enabling verification of triterpene composition in raw materials, extracts, and finished products. However, its focus on known compounds highlights the need for untargeted metabolomics capable of detecting novel triterpenoids. For instance, Adotey et al. applied LC-MS and multivariate statistical analysis to distinguish *Ganoderma* species from Ghana’s Lower Volta River region [34]. Partial least squares–discriminant analysis (PLS-DA) of total ion chromatograms (TICs) separated the samples into three species: *G. enigmaticum*, *G. resinaceum*, and *G. weberianum–sichuanense*. Heatmap visualization further revealed interspecific differences in metabolite composition, suggesting potential variation in bioactivity. This study illustrates the dual utility of metabolomics for both taxonomic resolution and therapeutic evaluation.

The metabolite profile of *Ganoderma* changes significantly with developmental stage [26]. A recent metabolomics–proteomics study of *G. lucidum* demonstrated that triterpenoid content, including ganoderenic acids E, H, and I, is highest at the budding stage, followed by a decline through maturation, implying that harvest timing critically influences therapeutic compound yield [35]. Earlier work by Chen & Chen (2003) further characterized multiple ganoderic acids (A, C, D, E, G) and ganoderenic acid D in *G. tsugae* fruiting bodies using HPLC and NMR, reinforcing the link between developmental stage and triterpenoid accumulation [36].

Zhang et al. employed ultra-high performance liquid chromatography coupled with Orbitrap high-resolution mass spectrometry (UPLC–Orbitrap–HRMS) to comprehensively profile metabolites in *G. lucidum* [37]. Their analysis identified 95 compounds, including ganoderic acids (i.e., A, B, C2, D, H, Y), ganoderenic acids (i.e., A, D, G), and additional bioactives such as kaempferol, genistein, and ergosterol. These compounds have been associated with a range of pharmacological activities, including antioxidant, antiinflammatory, and anticancer properties [38,39,40,41,42]. This integrative approach supports structure–activity relationship studies and the development of standardized therapeutic formulations.

Expanding the geographic scope of metabolomics, Wongkhieo et al. analyzed mycelial extracts of wild *G. australe* collected in Thailand using LC-MS/MS [43]. Their chemical profiling revealed the presence of lovastatin plus tentative identification of ρ-coumaric acid, nicotinamide, γ-aminobutyric acid (GABA), choline, nucleosides, amino acids, and saccharides. The detection of lovastatin, a known cholesterol-lowering compound, highlights the pharmaceutical and functional food potential of this underexplored *Ganoderma* species.

In a follow-up study, Adotey et al. examined *Ganoderma* mycelial cultures from Ghana and identified four lanostanoid triterpenes, ganoderenic acid A, ganoderenic acid D, ganoderic acid C6, and ganoderic acid G, through LC-MS-based dereplication [34]. Two additional compounds, ganoderic acid AM1 and ganoderenic acid K, were tentatively identified based on retention time and MS fragmentation. These findings suggest that local *Ganoderma* strains may possess distinct metabolic signatures, offering opportunities for region-specific product development.

Indeed, metabolomic profiling has become integral to the chemical characterization and quality assurance of *Ganoderma* products. Techniques such as LC-MS and NMR not only facilitate the quantification of known compounds but also support the discovery of novel metabolites. When integrated with taxonomic and ecological data, metabolomics enhances product standardization, species authentication, and pharmacological validation. Future efforts should prioritize untargeted analyses and expanded geographic sampling to fully explore the therapeutic potential of this medicinal genus.

### 5.3. Polysaccharides of Ganoderma

Polysaccharides represent one of the most extensively investigated classes of bioactive molecules in *Ganoderma* and are widely considered a major contributing factor to its medicinal properties [44]. In contrast to triterpenes, which are secondary metabolites with clear species- and stage-specific variation, *Ganoderma* polysaccharides are predominantly high molecular weight macromolecules associated with primary metabolism. These compounds are typically isolated from fruiting bodies, spores, or cultured mycelia and occur as β-glucans, heteropolysaccharides, or polysaccharide–protein complexes.

The most commonly reported polysaccharides in *Ganoderma* are β-D-glucans characterized by their β-(1→3)-linked backbones with varying degrees of β-(1→6) branching. However, significant structural diversity has been observed across species and strains, as well as in response to cultivation conditions and developmental stage [45]. In addition to homopolysaccharides, several heteropolysaccharides containing glucose, galactose, mannose, xylose, and fucose have also been reported. Several variations in monosaccharide composition, molecular weight, and branching architecture are reported to influence the solubility and biological activity, complicating direct comparisons between studies [46].

*Ganoderma* polysaccharides are strongly associated with their ability to impact innate immunity. Previous studies have shown that *Ganoderma* polysaccharides can promote macrophage activation, upregulate cytokine production, and promote natural killer (NK) cell and T-cell responses. These effects are mediated through the engagement of pattern recognition receptors such as dectin-1 (*CLEC7A*) and toll-like receptor 4 (TLR-4) on antigen-presenting cells [47,48,49].

Significant challenges are encountered in polysaccharide analysis that are not typically observed in small molecule analysis. For example, most LC–MS-based platforms used for triterpene profiling are generally unsuitable for intact polysaccharide analysis due to their high molecular weight and poor ability to be ionized. For this reason, many polysaccharide studies of *Ganoderma* have relied on water extraction followed by ethanol precipitation and chromatographic separation. Structural characterization is usually carried out through a combination of monosaccharide composition analysis, methylation analysis, Fourier-Transform Infrared (FTIR) spectroscopy, and NMR spectroscopy. Although these approaches produce important structural information, differences in extraction and purification methods can result in considerable variability between studies [46].

Notwithstanding, recent methodological advances have begun to improve the resolution of polysaccharide analysis. High-field NMR, size-exclusion chromatography coupled with multi-angle laser light scattering, and MALDI–TOF mass spectrometry have enabled more precise determination of molecular weight distributions and structural features [50]. In parallel, transcriptomic and genomic investigations have identified genes involved in polysaccharide biosynthesis, including glycosyltransferases and enzymes involved in nucleotide sugar metabolism. These findings provide a foundation for linking genetic variation to polysaccharide structure and bioactivity, although such integrative approaches remain limited within the genus [51].

Despite their recognized importance, polysaccharides remain underrepresented in comparative and regionally focused studies of *Ganoderma*, particularly in sub-Saharan Africa. Existing work from the region has largely emphasized antioxidant capacity or crude extract activity, with relatively little emphasis on detailed polysaccharide characterization. Incorporating polysaccharide-focused analyses into broader metabolomic and phylogenetic frameworks would provide a more complete understanding of chemical diversity within *Ganoderma* and support efforts toward species-specific pharmacological evaluation and product standardization [52,53].

## 6. Integrative Discussion and Regional Perspectives

Research on *Ganoderma* in Africa remains fragmented and uneven, with most studies concentrated in a small number of countries and often limited to morphology-based identification or single-marker analyses. Existing molecular investigations from West, Central, and North Africa collectively indicate that African *Ganoderma* diversity is underrepresented in global phylogenies and poorly integrated into current taxonomic frameworks. Most African species reports rely on macroscopic traits, with relatively few studies incorporating ITS sequencing and even fewer applying multilocus phylogenetic approaches or comprehensive metabolomic profiling. As a result, African *Ganoderma* taxa are frequently assigned to broadly defined species complexes established using Asian or European reference material, limiting confidence in species boundaries and obscuring regional diversity patterns. In contrast, *Ganoderma* research in East Asia, particularly in China and Japan, has benefited from extensive multilocus phylogenetic datasets, curated reference genomes, and large-scale metabolomic surveys. Asian taxa such as members of the *G. lucidum* complex have been resolved using ITS in combination with EF1-α, RPB2, and nLSU, revealing cryptic speciation and well-supported clades that are now widely accepted. Parallel metabolomic studies from Asia have established detailed triterpene and polysaccharide profiles that serve as benchmarks for chemotaxonomy, quality control, and pharmacological evaluation. By comparison, African *Ganoderma* species have rarely been analyzed using comparable molecular or metabolomic depth, making direct cross-regional comparisons difficult and precluding robust assessment of whether African taxa possess distinct evolutionary lineages or chemical signatures. Nevertheless, preliminary ITS-based phylogenies and limited LC-MS metabolomic analyses from Ghana and Cameroon suggest that African *Ganoderma* isolates may occupy underrepresented phylogenetic positions and exhibit metabolite profiles that do not fully overlap with well-characterized Asian taxa, highlighting the likelihood of region-specific diversity that remains insufficiently explored.

The integration of molecular systematics, phylogenetics, and metabolomics has significantly advanced the study of *Ganoderma*, enabling more accurate species delimitation, discovery of cryptic taxa, and detailed profiling of pharmacologically relevant metabolites. While morphology-based taxonomy laid the groundwork, it cannot meet the demands of taxonomic resolution, pharmaceutical validation, or product authentication. Molecular tools, especially ITS barcoding [14] and robust multilocus analysis using EF1-α and RPB2 alongside ITS and nLSU provide essential accuracy for species-level identification [12].

ITS has been widely adopted as the primary fungal barcode due to its high interspecific variability, amplification success, and broad representation in public databases [14]. However, ITS alone often fails to resolve closely related *Ganoderma* species; supplementary markers such as EF1-α, RPB2, and nLSU significantly improve phylogenetic resolution and reduce misidentification [12]. Multilocus approaches have revealed cryptic species and enabled taxonomic revision, with implications for conservation, biotechnology, and pharmacognosy.

Phylogenetic analyses have revealed geographically structured clades within *Ganoderma*, underscoring the importance of regional molecular studies for mapping global diversity [12]. Yet, substantial taxonomic uncertainty remains in Africa, where many species, such as *G. resinaceum* and *G. weberianum* are still identified solely by morphological features. Expanding the application of molecular barcoding and multilocus phylogenetics in African contexts will enhance our understanding of *Ganoderma* evolution and distribution while supporting the sustainable development of endemic fungal resources.

Concurrently, metabolomics has emerged as a powerful tool for characterizing *Ganoderma* bioactive molecules. Triterpenes, polysaccharides, nucleosides, and other secondary metabolites, linked to immunomodulatory, anti-inflammatory, and anticancer effects, have been comprehensively profiled using LC-MS, LC-MS/MS, and NMR in multiple species [34,43]. This biochemical diversity reinforces the therapeutic and commercial potential of *Ganoderma*, especially strains from underexplored regions.

Beyond their utility as chemotaxonomic markers, *Ganoderma* triterpenes, particularly ganoderic and ganoderenic acids, have been extensively linked to anticancer activity through multiple, partially overlapping molecular mechanisms. Several lanostanoid triterpenes, including ganoderic acids A, C, D, and H, have been shown to induce apoptosis in cancer cells via mitochondrial pathways, characterized by loss of mitochondrial membrane potential, cytochrome c release, and activation of caspase-9 and caspase-3 [54]. In parallel, these compounds frequently suppress pro-survival signaling by inhibiting the PI3K/Akt and MAPK pathways, resulting in reduced proliferation and enhanced apoptotic sensitivity [55,56,57].

Additional studies have demonstrated that ganoderic acids can exert anti-metastatic effects by downregulating matrix metalloproteinases (i.e., MMP-2 and MMP-9) and inhibiting epithelial–mesenchymal transition (EMT), thereby limiting tumor invasion and migration [54]. Anti-angiogenic activity has also been reported, with suppression of VEGF expression and interference with hypoxia-inducible factor-1α (HIF-1α) signaling [58,59]. Collectively, these mechanisms position *Ganoderma* triterpenes as multi-target modulators of tumor growth, invasion, and survival, supporting their relevance for pharmacological evaluation and reinforcing the importance of metabolomic profiling for identifying bioactive compound signatures.

Metabolite profiles vary with species, developmental stage, and cultivation conditions. For example, triterpenoid accumulation peaks during the primordia or budding stage and declines in mature fruiting bodies [35]. Regional metabolomics research, such as that conducted in Ghana, revealed distinct interspecific profiles among local strains, suggesting geography-dependent pharmacological variation [34].

Despite these advances, several critical challenges remain. First, the limited availability of complete genome sequences and curated metabolomic datasets for many *Ganoderma* species hampers the development of integrative identification frameworks. Second, the synergistic or antagonistic interactions among multiple bioactive compounds in crude extracts are not well understood. Although individual metabolites have demonstrated therapeutic potential, their combined effects on efficacy and toxicity require systematic study using integrated “omics” platforms and functional assays.

Third, variation in cultivation, extraction, and analytical protocols continues to impede product standardization. Environmental factors such as substrate composition, humidity, and light exposure can substantially influence metabolite production. Future work should prioritize the optimization and harmonization of these variables to ensure reproducibility and commercial quality control.

Remaining challenges include limited available genomes and curated metabolite datasets, uncharted synergy among bioactive compounds in crude extracts, and inconsistency in cultivation and analytical protocols affecting reproducibility and quality control. In particular, sub-Saharan African *Ganoderma* research is often descriptive and lacks molecular or chemical validation. Coordinated genomics and metabolomics efforts in Africa could uncover novel bioactive molecules and foster medicinal innovation. Long-term investment and international collaborative networks are essential to build research capacity. Addressing the current gaps in molecular and metabolomic characterization of *Ganoderma* in sub-Saharan Africa will require coordinated, practical strategies that leverage both regional biodiversity and emerging analytical capacity. Indeed, priority should be given to systematic field sampling combined with molecular barcoding, using ITS as a primary marker complemented by multilocus sequencing (i.e., EF1-α and RPB2) for representative collections. This approach would enable accurate species delimitation and reduce reliance on morphology-based identification.

Additionally, regionally focused metabolomic profiling should be integrated with taxonomic studies. Targeted LC-MS workflows for triterpenes and standardized polysaccharide extraction protocols can provide baseline chemical fingerprints for African *Ganoderma* taxa, facilitating comparison with well-characterized Asian and European species. Even limited datasets would substantially improve current knowledge of regional chemical diversity.

Likewise, the development of regional reference collections and sequence databases, including voucher-backed herbarium specimens linked to DNA sequences and metabolomic data, would enhance reproducibility and long-term accessibility. Such datasets could be generated through intracontinental collaborations, leveraging existing infrastructure while building local capacity.

Finally, integrating molecular and metabolomic data into phylogenetic and chemotaxonomic frameworks will support species-specific pharmacological evaluation, quality control, and future product standardization. Collectively, these strategies provide a practical roadmap for advancing *Ganoderma* research in underexplored African regions while aligning with global best practices in fungal systematics and natural product research.

## 7. Conclusions and Future Directions

The integration of molecular identification, phylogenetic analysis, and metabolomic profiling has significantly advanced the study of *Ganoderma*, transitioning it from a genus primarily defined by morphology to one characterized by genomic and biochemical precision. Advances in DNA barcoding, particularly ITS and multilocus sequencing have enabled more accurate species delimitation and revealed substantial cryptic diversity. Concurrently, metabolomic analyses have provided detailed insights into the chemical complexity that underlies the pharmacological properties of *Ganoderma*.

In this review, we have attempted to synthesize these interdisciplinary developments and propose a unified framework for taxonomic classification, biochemical characterization, and quality control of *Ganoderma* species. It underscores the essential role of molecular tools in overcoming the limitations of traditional taxonomy and highlights the utility of metabolomics in supporting both pharmacological validation and commercial standardization.

Despite substantial progress, key challenges remain, particularly in underexplored regions such as sub-Saharan Africa, where species diversity is insufficiently documented and largely unsupported by molecular or metabolomic data. Future efforts should prioritize the comprehensive genomic and metabolomic profiling of regional *Ganoderma* strains, the implementation of standardized analytical workflows, and the investigation of compound interactions that contribute to bioactivity.

Advancing these priorities through integrated, multidisciplinary research will facilitate a more complete understanding of *Ganoderma* as a medicinal resource, supporting biodiversity conservation, scientific innovation, and the development of evidence-based therapeutic applications.

## Figures and Tables

**Table 1:** Comparison of molecular markers commonly used for species delimitation and phylogenetic analysis in *Ganoderma*.

Molecular Marker	Typical Taxonomic Resolution	PCR Amplification Success	Clade-Specific Utility in *Ganoderma*	Key Limitations	Representative References
ITS (ITS1–5.8S–ITS2)	High at species level; limited for closely related taxa	Very high (universal primers; broad database coverage)	Effective for most *Ganoderma* species; insufficient for resolving species complexes (e.g., *G. lucidum* complex)	Intragenomic variation; poor resolution in cryptic lineages	[12,19,20]
ITS1	Moderate–high; improved geographic and intraspecific resolution	High	Useful for population-level and geographic clustering within species complexes	Limited phylogenetic depth; alignment challenges	[21,22]
ITS2	Moderate; short barcode with discriminatory potential	Variable; primer-dependent	Useful for rapid species identification and product authentication	Lower resolution for closely related species; inconsistent amplification	[23,24]
nLSU (28S rRNA)	Low–moderate; higher-level phylogeny	High	Useful for resolving deeper clades and subgeneric relationships	Insufficient for species-level delimitation	[16,20]
nSSU (18S rRNA)	Low; deep phylogenetic relationships	Very high	Useful for higher-order taxonomic placement	Highly conserved; limited species resolution	[13]
EF1-α (tef1α)	High; strong species-level resolution	Moderate–high	Resolves cryptic species within *G. lucidum* and allied complexes	Primer universality limited; fewer reference sequences	[25]
RPB2	Very high; robust multilocus phylogenetics	Moderate	Excellent resolution of species complexes and deep nodes	Lower amplification success; labor-intensive	[16,25]
Multilocus (ITS + EF1-α + RPB2 ± nLSU)	Very high; best overall resolution	Moderate	Gold standard for resolving cryptic species and taxonomic revision	Higher cost and technical demands	[16,22,26]

**Table 2:** Comparison of metabolomic approaches used to characterize major bioactive compounds in Ganoderma lucidum and related species.

Metabolomic Approach	Major Compound Classes Detected	Representative Ganoderma Applications	Strengths	Limitations	Typical Use Case
HPLC–UV/HPLC–DAD	Triterpenes (ganoderic acids A, B, C, D, H, etc.)	Quantification of ganoderic acids in fruiting bodies and commercial products	High reproducibility; suitable for routine quality control; relatively low cost	Requires authentic standards; limited structural information	Targeted quantification and product standardization
LC–MS/LC–MS/MS	Triterpenes, sterols, nucleosides, small phenolics	Species discrimination; developmental-stage metabolomics; regional chemotyping	High sensitivity; broad metabolite coverage; supports dereplication	Limited suitability for intact polysaccharides; ion suppression effects	Comparative metabolomics; chemotaxonomy
UPLC–HRMS (Orbitrap/Q-TOF)	Comprehensive secondary metabolite profiles	Untargeted profiling of *G. lucidum* and related taxa; discovery of novel metabolites	High mass accuracy; untargeted discovery; supports multivariate statistics	Expensive instrumentation; complex data processing	Species differentiation; biomarker discovery
NMR Spectroscopy	Triterpenes, polysaccharides, primary metabolites	Structural elucidation; developmental-stage metabolic changes	Quantitative; non-destructive; structural clarity	Lower sensitivity; large sample requirements	Structure confirmation; metabolic fingerprinting
GC–MS (derivatized samples)	Volatile compounds, monosaccharides (after hydrolysis)	Monosaccharide composition of polysaccharides	High resolution for small polar compounds	Requires derivatization; not suitable for intact macromolecules	Polysaccharide composition analysis
SEC–MALLS	High-MW polysaccharides	Molecular weight distribution of β-glucans and heteropolysaccharides	Accurate MW determination; absolute measurement	Limited chemical specificity; requires purified polysaccharides	Polysaccharide characterization
MALDI–TOF MS	Polysaccharide fragments, oligosaccharides	Emerging use for polysaccharide profiling	Rapid analysis; minimal sample prep	Limited quantitative accuracy; matrix effects	Complementary polysaccharide analysis

## Data Availability

No new data were created or analyzed in this study. Data sharing is not applicable to this article.

## Abbreviations

The following abbreviations are used in this manuscript:

DNA	Deoxyribonucleic Acid
EF1-α	Translation Elongation Factor 1-Alpha
HPLC	High-Performance Liquid Chromatography
HRMS	High-Resolution Mass Spectrometry
ITS	Internal Transcribed Spacer
ITS1	Internal Transcribed Spacer 1 subregion
ITS2	Internal Transcribed Spacer 2 subregion
LC-MS	Liquid Chromatography–Mass Spectrometry
LC-MS/MS	Liquid Chromatography–Tandem Mass Spectrometry
MS	Mass Spectrometry
NMR	Nuclear Magnetic Resonance
NMIMR	Noguchi Memorial Institute for Medical Research
nLSU	Nuclear Large Subunit ribosomal RNA gene
nSSU	Nuclear Small Subunit ribosomal RNA gene
PCR	Polymerase Chain Reaction
PLS-DA	Partial Least Squares–Discriminant Analysis
rDNA	Ribosomal Deoxyribonucleic Acid
rRNA	Ribosomal Ribonucleic Acid
RPB1	RNA Polymerase II Subunit 1
RPB2	RNA Polymerase II Subunit 2
TIC	Total Ion Chromatogram
UPLC	Ultra-Performance Liquid Chromatography
UPLC–Orbitrap–HRMS	Ultra-Performance Liquid Chromatography–Orbitrap High-Resolution Mass Spectrometry
